# Structural changes in brains of patients with disorders of consciousness treated with deep brain stimulation

**DOI:** 10.1038/s41598-021-83873-y

**Published:** 2021-02-23

**Authors:** Marina Raguž, Nina Predrijevac, Domagoj Dlaka, Darko Orešković, Ante Rotim, Dominik Romić, Fadi Almahariq, Petar Marčinković, Vedran Deletis, Ivica Kostović, Darko Chudy

**Affiliations:** 1 Department of Neurosurgery, Dubrava University Hospital, Avenija Gojka Suska 6, 10000 Zagreb, Croatia; 2 Centre of Excellence for Basic, Clinical and Translational Neuroscience, Croatian Institute for Brain Research, University of Zagreb, School of Medicine, Zagreb, Croatia; 3 Department of Neurosurgery, Sestre milosrdnice University Hospital Centre, Zagreb, Croatia; 4 Albert Einstein College of Medicine, New York, USA; 5 Department of Surgery, University of Zagreb, School of Medicine, Zagreb, Croatia

**Keywords:** Brain injuries, Diseases of the nervous system

## Abstract

Disorders of consciousness (DOC) are one of the major consequences after anoxic or traumatic brain injury. So far, several studies have described the regaining of consciousness in DOC patients using deep brain stimulation (DBS). However, these studies often lack detailed data on the structural and functional cerebral changes after such treatment. The aim of this study was to conduct a volumetric analysis of specific cortical and subcortical structures to determine the impact of DBS after functional recovery of DOC patients. Five DOC patients underwent unilateral DBS electrode implantation into the centromedian parafascicular complex of the thalamic intralaminar nuclei. Consciousness recovery was confirmed using the Rappaport Disability Rating and the Coma/Near Coma scale. Brain MRI volumetric measurements were done prior to the procedure, then approximately a year after, and finally 7 years after the implementation of the electrode. The volumetric analysis included changes in regional cortical volumes and thickness, as well as in subcortical structures. Limbic cortices (parahippocampal and cingulate gyrus) and paralimbic cortices (insula) regions showed a significant volume increase and presented a trend of regional cortical thickness increase 1 and 7 years after DBS. The volumes of related subcortical structures, namely the caudate, the hippocampus as well as the amygdala, were significantly increased 1 and 7 years after DBS, while the putamen and nucleus accumbens presented with volume increase. Volume increase after DBS could be a result of direct DBS effects, or a result of functional recovery. Our findings are in accordance with the results of very few human studies connecting DBS and brain volume increase. Which mechanisms are behind the observed brain changes and whether structural changes are caused by consciousness recovery or DBS in patients with DOC is still a matter of debate.

## Introduction

Human consciousness is often described as a complex phenomenon, consisting of two components, wakefulness and awareness^1,2^. While wakefulness is associated with functional brainstem neurons i.e. the reticular system projecting to both thalamic and cortical neurons, awareness is mostly related to the functional integrity of the thalamus, cerebral cortex, and their connections^1–4^.

Disorders of consciousness (DOC) occur as a result of interference with the mentioned systems. DOC can be acute and reversible, as a transient stage in a spontaneous recovery due to trauma, or chronic and irreversible, as in patients with unresponsive wakefulness syndrome (UWS)^1–4^. Prolonged DOC is not only long-lasting, serious, and currently untreatable, but also has severe consequences on a patient’s quality of life^5–7^. These conditions impose a serious consideration from both medical and ethical perspectives^7–9^. Clinical conditions such as anoxic or traumatic brain lesions can cause diffuse neuronal damage resulting in serious disorders ranging from minimally conscious state (MCS), UWS, coma, locked-in syndrome, and even brain death^1,10^. The boundaries separating the mentioned conditions are unclear, with each one including severities^1,2^. UWS is currently described as wakefulness without consciousness, including a complete lack of reactions. Additionally, it is characterized by variable cycles of sleeping and wakefulness, with preserved spontaneous respiration, digestion, and thermoregulation^1,2,10–13^. MCS is on the other hand characterized by inconsistent, but visible evidence of consciousness^1,14^ with the patient being awake but unaware. A patient in MCS can follow instructions occasionally, give simple gestural or non-verbal yes/no responses, and show a certain level of purposeful movement. The incidence of UWS is roughly 5 to 25 per million, while the prevalence in the adult population in the USA ranges from 40 to 168 per million^15^. Additionally, various neurological impairments occur, including seizures, movement disorders, myoclonus, focal motor, and sensorimotor deficits, as well as emotional, behavioral, and cognitive disturbances^16,17^.

Despite advances in diagnosis, improved classification, use of modern technology, and well-known pharmacological treatments alongside non-invasive brain stimulation, the surgical therapeutic approaches are still not significantly advanced^18,19^. Several studies used deep brain stimulation (DBS) of certain nuclei, such as centromedian parafascicle (CM-pf) complex and brain stem reticular formation, to regain consciousness in UWS and MCS patients^5,6,19–23^. Research thus often focused on the thalamus as a functionally important thalamocortical link, where stimulation through key thalamocortical and thalamus-forebrain circuitry was shown to be important and caused widespread effect^24^. The use of the CM-pf complex as a target was based on literature overview and clinical recommendation, as well as due to the connectivity and prospective effect on systems involved in consciousness^5,6,19–23,25^. Previous studies were done on a small cohort, mainly as case reports, and did not yield a definite answer whether this highly specific and refined method can be therapeutically successful^5,6,19–23,26–30^. Even though some studies claimed that there was no definitive evidence of the efficacy of this method for treatment of DOC patients, the usefulness of DBS was shown using controlled studies, as well as our previous study^6,22,23^.

Virtually all of the studies mentioned are missing an explanation of the detailed structural and functional cerebral changes induced by DBS. Recently, renewed interest in the grading of DOC and different trials of therapeutic approaches have been noted. Previous studies observed short-term volume changes in the human brain structure following DBS, potentially indicating restorative possibilities in patients with neurodegenerative diseases^31^. Modern magnetic resonance imaging (MRI) techniques play an important role in diagnostic classification and evaluation of cerebral damage in order to improve the gradation of different types and levels of DOC^32^. Moreover, MRI has been shown to be a potential tool for morphometric measurements and changes in a restructured, “reorganized” brain in DOC patients^33,34^. Modern neuroimaging thus uses different methods to objectively evaluate both structural and functional changes as an effect of different treatments.

Previously, it has been documented that DBS changes function in such structures as the striatum, hippocampus, amygdala, etc. It would thus arguably follow that if electrical stimulation changes the brain’s structure and its function, stimulation of damaged circuitry could then induce both functional and structural changes with an eventual improvement of function^35–37^.

To determine the impact of CM-pf stimulation on a possible brain structural reorganization after functional recovery, we performed a quantitative volumetric MRI analysis in DOC patients who underwent CM-pf DBS. Our study aimed to reveal if CM-pf DBS could induce an impact on brain structural reorganization in DOC patients and lead to a changed volume of specific cortical and subcortical structures.

## Methods

### Patients

This retrospective study included five patients who underwent CM-pf DBS due to DOC after which they achieved functional recovery (Table 1). Out of the five patients, three were female, average age 16 ± 1.53. The cause of injury in one patient was anoxic due to cardiac arrest, while other two patients experienced traumatic injuries. Two patients were male with an average age of 20 ± 4.24 years. In both of these patients the cause of injury was anoxia due to cardiac arrest. The length between the injury and time to DBS was 2 months in males and 11 ± 5.13 months (range 4–14) in females. The average follow-up duration in female patients was 88 ± 38.17 months (range 30–102), while in male patients it was 84 ± 38.18 months (range 57–111).

**Table 1:** Demographic and clinical characteristics of consciousness improved patients after DBS.

Patient no.	Sex	Etiology	Age at Injury (years)	Time from initial injury to DBS (month)	Level of awareness (RDR, C/NC scale)		Follow up duration (month)
					Before DBS	After DBS	
1	♂	CA	17	2	2.0/1, MCS	0, aware	111
2	♂	CA	23	2	1.8/1, MCS	0, aware	57
3	♀	TBI	15	11	1.6/1, MCS	0, aware	102
4	♀	CA	16	4	2.6/2, UWS	0, aware	88
5	♀	TBI	18	14	2.6/2, UWS	0, aware	23

*CA* cardiac arrest, *TBI* traumatic brain injury, *RDR* rappaport disability rating scale, *C/NC* coma/near coma scale, *UWS* unresponsive wakefulness syndrome, *MCS* minimally conscious state.

In the present study we included patients previously described in Chudy et al*.*^22,23^. Forty-nine patients were enrolled in the previous study. Fourteen patients (10 UWS and 4 MCS) fulfilled neurophysiologic, clinical, and neuroimaging criteria and underwent DBS implantation. Four patients (3 MCS and 1 UWS) achieved functional recovery, while other patients were further followed by an attending palliative physician^22,23^. In this study we included one additional UWS patient who underwent DBS implantation after fulfilling the aforementioned criteria and achieved functional recovery.

Patients selected for DBS were evaluated utilizing the standard Rappaport Disability Rating (RDR) scale and the Coma/Near Coma (C/NC) scale^22,23,38^. Inconsistently responsive to simple commands patients were classified as MCS patients (C/NC Level 1), while patients who were not able to respond to any command were classified as UWS (C/NC Level 2–4). DOC patients were selected based on three main factors: their neurophysiologic evaluation, 12/24 h electroencephalography (EEG) and neuroimaging (MRI). The neurophysiological criteria we investigated were recordable somatosensory evoked potential (SEP), motor evoked potentials, and brainstem auditory evoked potentials, even with pathological parameters such as prolonged latencies or central conduction time. The entry criterion for SEPs was recordability via stimulation of median nerves, with or without SEPs elicited by tibial nerve stimulation. The second main factor we looked at was EEG. The entry criterion for this method was the presence of periods of desynchronized EEG activity during 12/24 h of monitoring processed EEG^22,23^. Obtained neuroimaging (MRI) showed absence of a structural defect. After passing extensive selection criteria, the patients underwent DBS electrode implantation into the CM-pf complex of the left thalamic intralaminar nuclei, while in patients with posttraumatic lesions, the electrode was placed in the better-preserved hemisphere^22,23^. On the third postoperative day, monopolar stimulation was initiated using the contact eliciting the strongest arousal response with minimal current (25 Hz frequency, 90 µs pulse duration, voltage 2.5–3.5 V). Stimulation was applied for a 30-min period every two hours during the daytime. Stimulation duration was up to 10 months after DBS electrode implantation. The informed parental/caregivers consent was obtained for all patients in accordance with the Declaration of Helsinki and ethical approval was obtained from the Institutional Review Board of the Dubrava University Hospital, School of Medicine, University of Zagreb, Croatia.

### MRI acquisition

**Figure 1:**
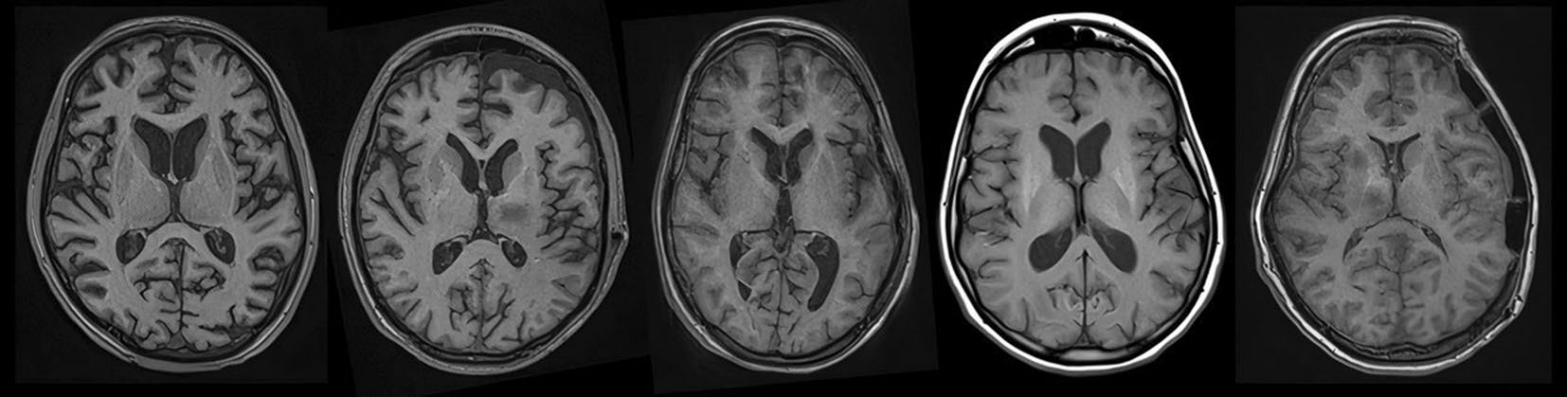
Initial structural MRI scans of included patients showing an absence of any large brain lesion (patients 1–5, from left to right).

MRI scans were obtained on a 1.5 T MRI scanner (MAGNETOM Aera, Siemens Healthineers, Erlangen) using 24 channeled head coils. Standard clinical sequences were used, as well as high-resolution 3D T1-weighted magnetization-prepared rapid acquisition gradient echo (MPRAGE) sequence with the following scanning parameters: TR = 2300 ms, TE = 3 ms, flip angle = 15°, matrix size = 256 × 256, field of view = 256 mm, voxel size = 1.0 × 1.0 × 1.0 mm. Obtained MRI scans were closely examined showing the absence of any large brain lesion (Fig. 1). MRI was obtained three times; the first one prior to DBS implantation, the second one between 8 and 12 months after surgery (approximately 1 year), and the third time between 1 and 7 years after DBS implementation.

### Quantitative volumetric methods

**Figure 2:**
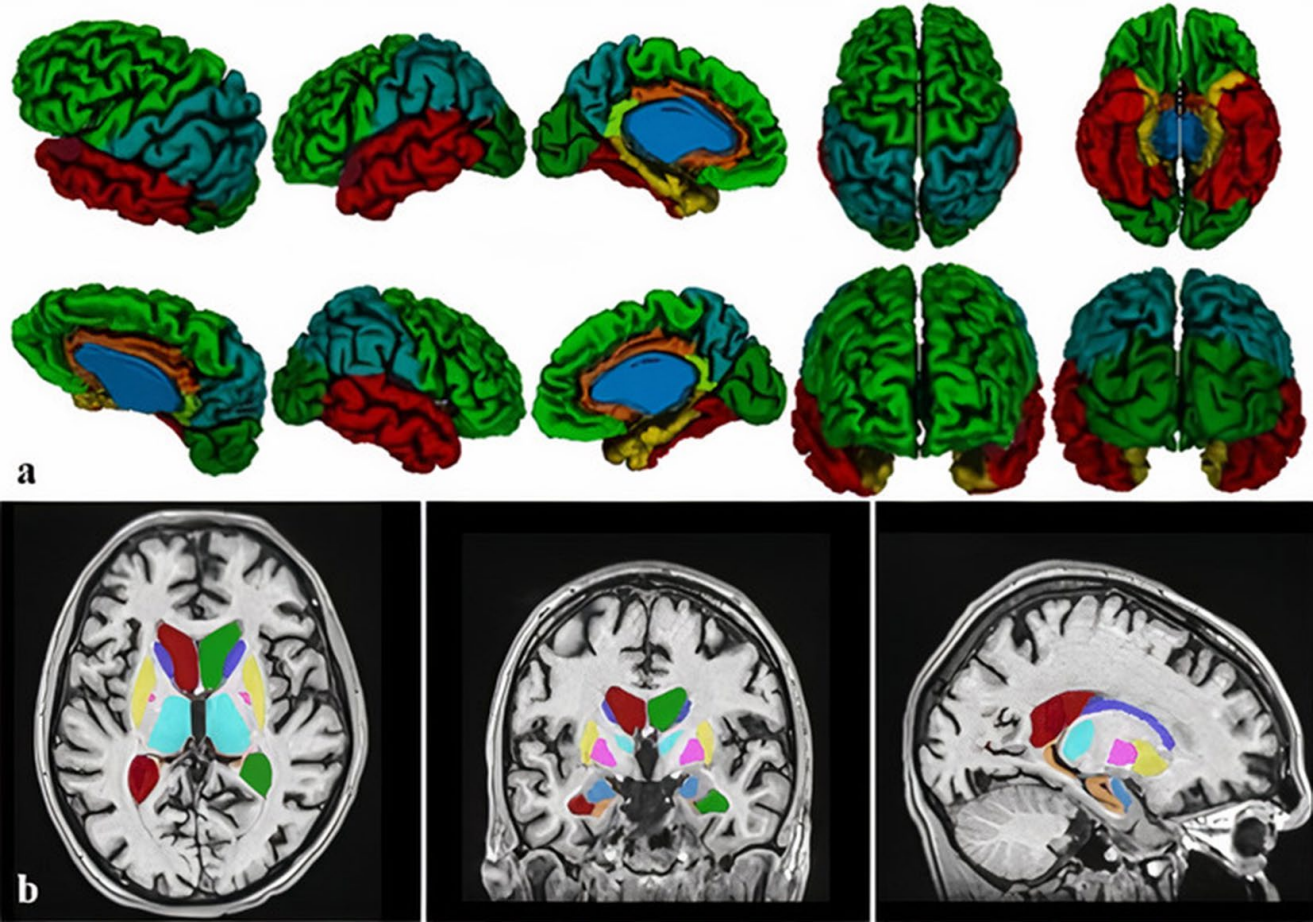
Volumetric analysis was conducted using the CIVET pipeline measuring regional cortical volume and thickness for parietal, occipital, frontal, temporal lobes, isthmus of the cingulate gyrus, parahippocampal, and cingulate gyrus and insula (**a**), and volBrain software, for subcortical structures segmentation (**b**). Single-subject volumetric analysis is presented.

Volumetric analysis was conducted using an automated CIVET processing pipeline (version 2.1.0., http://www.bic.mni.mcgill.ca/ServicesSoftware/CIVET) of different inter-dependent algorithms, on a web-based portal, CBRAIN (https://portal.cbrain.mcgill.ca), providing tools for observer-independent corticometric analysis^39–41^. During analysis, the pipeline included the following steps: correction of radiofrequency intensity and nonuniformity artifacts corrected using the N3 algorithm^42^, linear registration using the 9-parameter affine process of translation, scaling, and rotation, and the 3D T1 volume registration to the ICBM 152 stereotactic space^43,44^. The brain was masked, including scull stripping and tissue classification as grey matter, cerebrospinal fluid, and white matter, using the discrete classifier with advanced neural network methods^45,46^. Cortical, grey matter and white matter surfaces extraction using the Laplacian map, partial volume classification^47,48^ and thickness calculation^49^ were final steps. The surfaces were smoothed and registered in order to calculate the regional cortical volume and the average regional thickness of the cerebral cortex of automatically parcellated lobes^50–52^. Quantification of regional cortical thickness averages where lobe borders were determined by sulcal landmarks and detected by the CIVET pipeline and regional cortical volume estimates were made for parietal, occipital, frontal, temporal lobes, parahippocampal gyrus, cingulate gyrus, isthmus of the cingulate gyrus, and insula (Fig. 2a). Subcortical structure segmentation (putamen, caudate, thalamus, globus pallidus, hippocampus, amygdala, nucleus accumbens) was performed using an automated MRI brain volumetry system, volBrain^53^ (Fig. 2b). Volumetric analysis was performed at three measuring points, according to MRI scans: prior to DBS, approximately 1 year after, and 7 years after DBS implementation.

### Statistical analysis

Data analysis was performed using the MedCalc Statistical Software version 12.5.0 (MedCalc Software, Ostend, Belgium; https://www.medcalc.org). Data were plotted as individual values, horizontal lines and markers represent mean ± SD. Comparison of the regional cortical volumes and thicknesses of parcellated lobes and volumes of subcortical structures were obtained. Depending on the distribution and type of variables, volumetric data in all measuring points were analyzed using one-way ANOVA, with the Student–Newman–Keuls post hoc test, or Kruskal–Wallis test with pairwise comparisons according to Conover^54^. In order to reduce intersubject variability we have performed the regional volumes normalization by head size. Normalization was done using the subject with lowest head size value as a calibrator value to calculate the coefficient (subject value/calibrated value). Regional volumes were multiplied with the calculated coefficient for each subject and further analyzed using previously mentioned statistical test. We did not correct for multiple comparisons in the present small patient sample. The statistical significance was set at p < 0.05.

### Ethics approval and consent to participate

This study was carried out in accordance with the recommendations of the Ethical Board of the Dubrava University Hospital and University of Zagreb, School of Medicine with written informed parents/caregivers consent from all subjects in accordance with the Declaration of Helsinki. The protocol was approved by the Institutional Review Board of the University Hospital Dubrava and University of Zagreb, School of Medicine.

## Results

**Figure 3:**
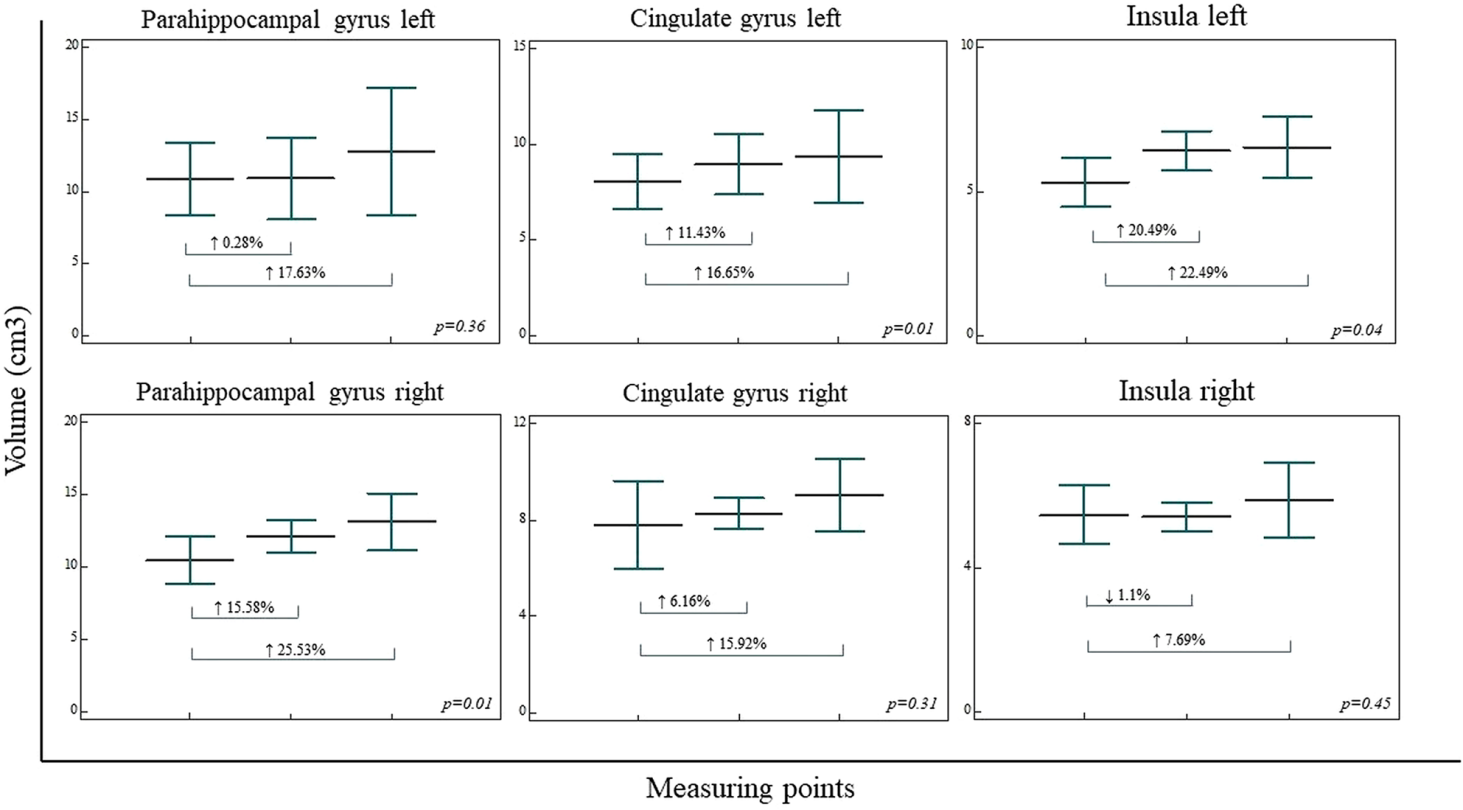
Regional cortical volumetric analysis in three measuring points (prior to DBS, 1 year after DBS, and 7 years after DBS). The regional cortical volumetric analysis revealed significant volume increase in right parahippocampal gyrus, left cingulate gyrus, and left insula, while the trend of volume increase was presented in left parahippocampal gyrus volume, right cingulate gyrus volume, and right insula volume. Vertical bars, standard deviation*.*

Analysis of segregated volumes of the lobes of the cerebral cortex in three measuring points revealed statistically significant volume increase in right parahippocampal gyrus (↑25.53%) (*F*(*2*,* 9*) = *6.658*,* p* = *0.01*), left cingulate gyrus (↑16.65%) (*F*(*2*,* 9*) = *6.679*,* p* = *0.01*) and left insula (↑22.74%) (*F*(*2*,*9*) = *6.202*,* p* = *0.04*)*.* However, volume increase was observed for left parahippocampal gyrus volume (↑17.63%) (*F*(*2*,*9*) = *1.142*,* p* = *0.36*), right cingulate gyrus volume (↑15.92%) (*F*(*2*,*9*) = *1.311*,* p* = *0.31*) and right insula volume (↑7.69%) (*F*(*2*,*9*) = *0.871*,* p* = *0.45*) (Fig. 3). Volumetric analysis of frontal (L ↓7.57%, R ↑3.22%), parietal (L ↑0.05%, R ↓2.54%), occipital (L ↓9.99%, R ↓15.08%,), and temporal (L ↓3.38%, R ↑2.14%) lobe, as well as isthmus of the cingulate gyrus (L ↑1.53%, R ↑1.52%), bilaterally, did not revealed statistically significant results.

**Figure 4:**
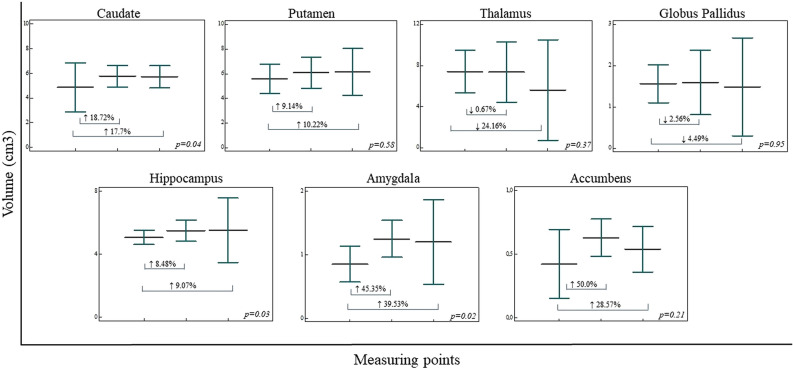
Subcortical structures volumetric analysis in three measuring points (prior to DBS, 1 year after DBS, and 7 years after DBS). Significant volume increase of caudate, hippocampus, and amygdala were presented, while putamen and accumbens volumes presented a trend of volume increase during three measuring points. Both globus pallidus and thalamus volume decreased over time. Vertical bars, standard deviatio*n.*

Subcortical structures volumetric analysis revealed statistically significant volume increase in caudate (↑17.7%) (*F*(2,9) = *4.964*,* p* = *0.04*), hippocampus (↑9.07%) (*F*(2,9) = 4.052,* p* = 0.02) and amygdala(↑39.53%) (*F*(2,9) = 4.163,* p* = 0.02). While putamen (↑10.22%) (*F*(2,9) = 1.576,* p* = *0.58*) and accumbens (↑28.57%) (*F*(2,9) = 1.915,* p = 0.21) volumes* were observed increased, both globus pallidus (↓4.49%) (*F*(2,9) = 0.054,* p* = 0.95) and thalamus (↓24.16%) (*F*(2,9) = 1.104,* p* = 0.37) volume revealed volume decrease over time, especially prominent in thalamus (Fig. 4). While right parahippocampal gyrus regional cortical thickness increase significantly (↑16.9%) (*F*(2,9) = 1.937,* p* = 0.02), regional cortical thickness increase was observed in both right cingulate gyrus and insula bilaterally, as well as in the left parahippocampal gyrus, although these values were not statistically significant. Regional cortical thickness is observed to decrease over time in the parietal, frontal, temporal, and occipital lobe, as well as the isthmus of the cingulate gyrus.

Statistical analysis of normalized segregated volumes revealed significantly higher volume of the left insula (*F*(2,9) = 6.804,* p* = 0.01). Volumes of the right insula (*F*(2,9) = 1.407,* p* = 0.29), the left parahippocampal gyrus (*F*(2,9) = 1.142,* p* = 0.36), the right parahippocampal gyrus (*F*(2,9) = 1.694,* p* = 0.23), the left cingulate gyrus (*F*(2,9) = 3.064,* p* = 0.09) and the right cingulate gyrus (*F*(2,9) = 2.423,* p* = 0.14), as well as volumetric analysis of frontal, parietal, occipital, and temporal lobe, and isthmus of the cingulate gyrus, did not significantly differ among groups. Subcortical normalized volumes of the amygdala (*F*(2,9) = 4.451,* p* = 0.04) were significantly higher. Normalized volumes of the caudate (*F*(2,9) = 2.952,* p* = 0.22), hippocampus (*F*(2,9) = 3.390,* p* = 0.08), putamen (*F*(2,9) = 1.015,* p* = 0.40), accumbens (*F*(2,9) = 2.436,* p* = 0.14), globus pallidus (*F*(2,9) = 0.032,* p* = 0.96) and thalamus (*F*(2,9) = 0.747,* p* = 0.50) did not significantly differ.

All five patients included in the study raised to full awareness and regained the ability to communicate and interact. Two male patients (Patient 1 and 2) were graded as MCS using RDR and C/NC scale (Table 1). Both of them are currently able to live independently to a large extent^22,23^, although both of them are experiencing problems with concentration and memory (Table 2). Out of the three female patients, one was graded as MCS (Patient 3), while two of them were graded as UWS (Patient 4 and 5) using RDR and C/NC scale (Table 1). All female patients regained consciousness, although they are currently still unable to live independently^22,23^. The main obstacle for further progress is related to motor difficulties, such as spasticity, contractures, inability to move their limbs, to articulate words, etc. For several years, their communication was in a form of non-verbal signs, predominantly as facial gestures and/or limb movement. Today, these patients are able to form simple words (Table 2).

**Table 2:** Neurocognitive and behavioral assessment of consciousness improved patients after DBS.

Patient no.	Sex	Etiology	DOC	Verbal communication	Non-verbal communication	Observed physical difficulties
1	♂	CA	MCS	+	+	Stuttering, problems with concentration
2	♂	CA	MCS	+	+	Slowness in movements and talk; difficulties in fine motor skills; problems with concentration
3	♀	TBI	MCS	+ *	+	motor difficulties (spasticity, contractures, inability to move limbs and to articulate words)
4	♀	CA	UWS	+ *	+	motor difficulties (spasticity, contractures, inability to move limbs and to articulate words)
5	♀	TBI	UWS	+ *	+	motor difficulties (spasticity, contractures, inability to move limbs and to articulate words)

*CA* cardiac arrest, *TBI* traumatic brain injury, *UWS* unresponsive wakefulness syndrome, *MCS* minimally conscious state.

*Under intensive speech therapy at the moment, able to pronounce simple words.

## Discussion

Previous studies reported that DBS in DOC is still in its exploratory phase^5,6,19–23,26–30^. Keeping in mind the ethical criteria and seriousness of DOC, our previous results showed limited but encouraging success^22,23^. Although brain atrophy was initially observed on MRI prior to DBS, it was difficult and challenging to explain the volume increase of several brain structures and areas after DBS, which was in accordance with previous studies^31^. Two main aspects are going to be discussed in the following paragraphs. Firstly, a brief overview of consciousness, the role of the thalamus in conscious networks, as well as the general functional effects of stimulation. Secondly, we will discuss the structural changes that occurred after functional improvement, as a result of processes of repair, plasticity, and reorganization.

### General effects of deep brain stimulation

All major factors and phenomena of consciousness are not fully understood at the moment. Despite numerous different study explanations and well-elaborated hypotheses, the neurobiological mechanism of consciousness is still largely unexplored^37,55–58^. What is known however is that various components of the central nervous systems participate simultaneously in this intriguing phenomenon^56–61^. Alongside the reticular ascending systems projecting from the upper reticular formation of the brainstem, other structures participate significantly in forming consciousness. These structures include monoaminergic pathways, the locus coeruleus, serotonergic and dopaminergic systems, the forebrain cholinergic system, as well as the nucleus basalis^62–66^. In addition, a special system of neurons connecting the brainstem to the interstitial neurons may also participate^67^ and form important networks for maintaining and regulating cortical functions^68,69^. These neurons project towards a number of subcortical structures, in which projections from thalamus nuclei are also received^62–66^. We propose that the main functional system responsible for the observed structural changes is widespread thalamocortical and thalamosubcortical (feedback) circuitry, modulated by several systems: modulatory monoaminergic systems, the reticular ascending system from the upper brainstem, basal forebrain cholinergic systems, etc.

The thalamus is a crucial relay of the cortico-striatal-thalamo-cortical circuits and its role in arousal, attention to salient stimuli, and processing of information has been documented by both animal models as well as functional connectivity studies in humans^24,25,70–74^. The CM-pf complex as the main part of the intralaminar nuclei^24^ provides particularly strong connections to several subcortical structures^74^. It also provides strong connections to the basal ganglia^75^, primarily the caudate nucleus^76^, putamen^24^, nucleus accumbens^77^, and pallidum, all of which was demonstrated using both tracing and tractographic studies^78^. A relatively weaker subcortical connectivity was noticed between CM-pf and the amygdala^79^, and the hippocampus^80,81^. On the other hand, excitatory projections from the CM-pf complex toward cortical areas have been reported as much weaker and non-specific^24^. The cortical projections seem to be limited to the agranular cortex with the only exception being the anterior insular/frontal operculum^78^. Additionally, the CM-pf complex probably interacts with various cortical areas via the corticostriatal pathways, through strong retrograde projections^36^.

The selection of the thalamus as a stimulation target thus seems justified because of its potent effect in the thalamocortical circuitry. Our preliminary results showed the thalamic volume decrease less than 1% at the second measuring point, while at the third measuring point thalamic volume decrease of almost 25% was observed (Fig. 4). Since the thalamic volume was slightly decreased at the period when functional recovery was observed in patients, we presume that the constant volume contributed to functional connectivity of the thalamus and previously mentioned structures. Results of positive stimulation effects point to the thalamus and reorganization of functional circuitry as important factors in recovery after DOC. The DBS electrode, positioned deep inside the brain, provides permanent stimulation. This in turn provides new possibilities for integrating function and structure for consciousness recovery. However, despite the evidence that thalamic intralaminar nuclei have an influence on cerebral function and connectivity, it is unlikely that it can explain such major changes in the brain structure volume. In our patients, a larger volume decrease was observed several years after the functional recovery, thus we can only assume it occurred as a further adaptation of the thalamus to the brain reorganization due to the initial brain injury, either trauma or anoxic lesion. Additionally, the thalamus volume could decrease in response to the lack of adequate stimulus. Once the DBS is removed, the stimulus that maintained thalamic volume by providing sufficient stimulation may cease. The variability of structural changes observed after DBS may be partly explained by the great variety of cortical, subcortical, and brainstem functional systems involved. This variability suggests that there is no single functional system affected. Therefore, besides the thalamus and indirectly affected systems, other factors play a possible role and will be discussed below.

### Structural effects observed on MRI

The exact underlying mechanisms contributing to volume changes are still unknown. There are several possible explanations about how long functional stimulation could have led to a volume increase. These include synaptogenesis, gliogenesis, axonal remodeling, micro vascularization, neuronal size increase, and extracellular matrix changes. A combination of all these factors is also possible. Of note is that adult neurogenesis has also been described in certain brain regions (i.e. the hippocampus)^31,82^. It is well known that after various lesions, the brain volume could be changed by different underlying processes of brain tissue repair (apoptosis, microstructural glial reactivity, changes in the extracellular matrix, etc.), plasticity (sprouting, myelination, dendritic plasticity), and reorganization^83–87^. Therefore, we could expect similar events occurring and leading to the increased brain volume in our patients. Additionally, processes of repair and plasticity probably overlap. The reorganization of pathways is thus expected to be primarily functional (since various pathways transduce electrical information when the brain is stimulated), rather than structural, which is possible primarily during development^86,87^. Nevertheless, considering how the most voluminous components of the brain tissue are cell bodies of neurons, glia, dendrites, and myelinated axons^88–90^, it is unlikely that these cells experience a significant increase in their size receiving more excitatory input. Moreover, an increase of dendritic branching postsynaptic spines of super excited neurons is possible. The data on glia are less consistent, but it is generally expected that astroglia follows a metabolic increase of its function and become hypertrophic when stimulated^91^. Volume increase could therefore occur during an increase in synapsis number and size which are more activate during prolonged stimulation of important excitatory glutamatergic neurons^92^. However, synapsis makes only a small proportion of the brain volume^90^.

Several examples of increased brain volume during development in abnormal conditions have been previously reported. In such cases, hyper-connectivity within the cortex was explained by the changes in the extracellular substance, which makes approximately 70% of the developing brain, while being scarce in the normal adult brain^93–95^.

The aforementioned structural changes (repair, plasticity, and reorganization) are impossible to follow in patients, even if *postmortem* material is available. Therefore, MRI is so far the only available method providing general information that something is structurally different. Additionally, it is especially useful to combine different MRI techniques such as tractography with easily performed volumetric measurements, a valuable indicator that structural processes are taking place in the brain after stimulation. Only a few volumetric MRI analyses in patients with DOC were performed so-far^33,34^. Grey matter volume of the parahippocampal gyrus, thalamus, and caudate were previously presented to be the key features differing healthy subjects and patients with DOC. Additionally, white matter volumes of the parahippocampal gyrus, isthmus of cingulate gyrus, and brainstem were previously described as the most affected white matter regions^34^. Analysis of the subcortical structures was previously performed only for the thalamus; slower atrophy of the thalamus was observed in MCS than in UWS patients^33^. These results suggest that patients with better-preserved levels of consciousness are more likely to preserve an increased brain volume over time. Our results indicate that the most informative region is the parahippocampal area. This area is known to be important for the formation of episodic memories as well as permitting rapid processing which in turn enables contextualization of outside events. It is therefore well located to bind associations from other cortical streams, suggesting an important function in consciousness^96^. Our findings are in the accordance with the results of previous preclinical studies of DBS in rodents which observed structural neuroplasticity (hippocampal neurogenesis^97,98^), as well as an increased complexity of apical dendrites and the length of basal dendritic trees of pyramidal neurons of the hippocampus^99^. Furthermore, our findings are in accordance with results showing short-term brain volume increase, namely the hippocampal area, in Alzheimer’s dementia patients following DBS^31^.

Several limitations of the presented study should be mentioned, such as a small number of patients, their mixed gender, different age and etiology of injury as well as different times for MRI scans from the initial injury and volumetric analysis in included patients. Still, we believe the presented results are valuable and can be a valid starting point for future research.

## Conclusions

Even on a small sample size included in the present study, we tried to emphasize the importance of morphometric MRI quantification which can expand our understanding of the relationship between brain structures and consciousness recovery. Our results are in accordance with the results of very few human studies connecting DBS and brain volume changes. They also support the idea that DBS in the CM-pf complex may have a widespread effect on cerebral function and structure.

Structures with altered volumes belong to cortico-thalamo-cortico-basal ganglia circuitry, limbic circuitry and at the same time have strong modulatory monoaminergic brainstem input. The nature of the initial lesion of neural networks, neurons, and glia, is difficult to determine with the current criteria and methodology applied. Which mechanisms are behind the observed brain changes and whether the observed structural changes are caused by consciousness recovery and/or DBS in DOC patients is yet to be elucidated. In future studies, different MRI volumetric analysis of cortical areas or deep brain structures could help to identify cell types and more details contributing to the regional volume change.

## Data Availability

The datasets generated for this study are available on request to the corresponding author.
